# Results of optimal debulking surgery with bowel resection in patients with advanced ovarian cancer

**DOI:** 10.1186/s12957-016-0800-1

**Published:** 2016-02-29

**Authors:** Pawel Derlatka, Jacek Sienko, Laretta Grabowska-Derlatka, Piotr Palczewski, Anna Danska-Bidzinska, Mariusz Bidzinski, Krzysztof Czajkowski

**Affiliations:** 2nd Department of Obstetrics and Gynecology, Medical University of Warsaw, Karowa 2 St, 00-315 Warsaw, Poland; 2nd Department of Clinical Radiology, Medical University of Warsaw, Warsaw, Poland; The Faculty of Health Science, The Jan Kochanowski University in Kielce, Kielce, Poland

**Keywords:** Advanced ovarian cancer, Bowel resection, Optimal debulking surgery

## Abstract

**Background:**

The surgical treatment of patients with advanced-stage ovarian cancer is based on maximal cytoreduction with widening the debulking on the extra-ovarian tissues and infiltrated organs. The purpose of the study was to assess the outcome after optimal cytoreduction with partial bowel resection and to find the risk factors of relapse. Another goal was the quantitative and qualitative assessment of intra- and postoperative complications in the studied group.

**Methods:**

The analysis of debulking procedures with intestinal resection and postoperative period in 33 ovarian cancer patients, The International Federation of Gynecology and Obstetrics (FIGO) stages III and IV, was performed.

**Results:**

The optimal cytoreduction defined as less than 1.0 cm residual disease was achieved in all patients including the following: 26 patients (78.8 %) with no macroscopic residual disease, 4 patients (12.1 %) with the largest residual tumor less than 0.5, and 3 patients (9.1 %) with 0.5 cm to less than 1.0 cm residual disease. The rectosigmoid resection was the most common surgical procedure (*n* = 27). The risk of relapse was significantly higher in subjects who had the macroscopic residual tumor left during the primary operation (57.1 vs. 11.5 %, *P* = 0.035). A primary bowel tumor size was another predictor of relapse. The maximum tumor diameter was significantly larger (14.9 ± 6.7 cm vs. 10.3 ± 4.7 cm, *P* = 0.047) in patients who developed the relapse.

**Conclusions:**

As presented in the article, our outcomes and other authors’ observations indicate that debulking surgery with bowel resection in patients with advanced ovarian cancer brings good results. Complications connected with bowel surgery are to be accepted. The interesting thing is that a primary bowel tumor size was a predictor of relapse.

## Background

Ovarian cancer incidence has been on a steady rise in recent years. In 2011 alone, 3257 new cases of ovarian cancer were diagnosed in Poland, of which 70 % were The International Federation of Gynecology and Obstetrics (FIGO) stage III and IV cancers. In the same year, 2558 ovarian cancer-related deaths were recorded [1]. In European women, ovarian cancer constitutes 4 % of cancers and is the sixth most common cause of cancer-related death [2].

The treatment of patients with advanced-stage ovarian cancer is based on surgery and adjuvant chemotherapy. The surgeon aims into achieving a maximal possible cytoreduction, while at the same time minimizing the trauma to the patient. The goal of surgery is *optimal* surgical cytoreduction, which is defined as residual disease of 10 mm or less [3]. Numerous studies have shown that the prognosis improves with the reduction of tumor volume at the end of surgery and that the patients in whom the removal of all grossly evident tumor (i.e., “complete” cytoreduction) was possible have the best prognosis [4–6]. In recent years, a trend to improve cytoreduction by performing surgery on the extra-ovarian tissues and organs infiltrated by the tumor has become apparent. A few series of extended surgical procedures including abdominal and diaphragmatic peritonectomy, partial liver resection, and partial pancreatectomy or splenectomy have been published. The resections of parts of the digestive tract, especially the large bowel, are increasingly commonly performed [7–9]. According to the authors of those reports, the risk associated with such procedures seems justified in view of an improved prognosis.

Even though this type of surgery is usually performed in expert gynecologic oncology centers, it seems to be associated with a higher rate of intraoperative and early postoperative complications such as bleeding, anastomotic dehiscence, and infection. In selected patients, ostomy becomes necessary, which inevitably leads to a significant deterioration of the comfort of living. At this point, it becomes debatable whether the advantages of optimal cytoreduction during primary treatment are sufficient to compensate for the risks of extended surgery.

The purpose of the study was to assess the outcome after optimal cytoreduction with partial bowel resection and to find the risk factors of relapse. Another goal was the quantitative and qualitative assessment of intra- and postoperative complications in the studied group.

## Methods

Between October 2010 and December 2013, 33 extended first-line surgeries with partial bowel resection were performed in patients with FIGO stage III and IV ovarian cancer. Multi-detector computed tomography (MDCT) or magnetic resonance imaging (MRI) with diffusion-weighted imaging were performed in all subjects during qualification for optimal cytoreduction to assess the extent of the tumor spread and likelihood of the necessity to perform a bowel resection. The exclusion criteria for primary surgery included tumor deposits covering a greater part of the parietal peritoneum and enteric surface, combined infiltration of the periaortic space and mesentery, and infiltration of the hepatic hilum. Patients meeting the exclusion criteria were referred for neoadiuvant chemotherapy (PCL + CBDCA) provided the diagnosis of cancer was confirmed by laparoscopic biopsy or the presence of the neoplastic cells in the peritoneal fluid taken during paracentesis. In the second situation, the CA125/CEA ratio was required to be more than 25. After three courses of chemotherapy, the patients underwent MDCT and were requalified to cytoreduction surgery. The presence of up to several limited peritoneal implants was not considered a contraindication to surgery. In equivocal cases, diagnostic laparoscopy was preformed shortly before planned surgery. One day before the surgery, the patients underwent bowel preparation with cathartics and cleansing enemas. Antibiotic and antithrombotic prophylaxis was administered in all patients. The final decision about performing bowel resection and about its extent was undertaken during the surgery.

In the descriptive analysis of the results, the following parameters were taken into account: a maximum diameter of the resected tumor, type of surgical procedure, perioperative blood loss, need for reoperation, postoperative complications including wound infection and fever >38 °C for longer than 48 h, hospital stay length, and time from surgery to the onset of chemotherapy.

The response to treatment and time to progression were used as clinical endpoints for this study. The patients with the relapse were compared with the subjects who had no relapse during the observation. The detailed characteristics of the patients are presented in Table 1.

**Table 1:** Patient characteristics

	All patients *N* = 33	No relapse group *N* = 26	Relapse group *N* = 7	*P*
Age (years ± SD)	58 ± 10.4	56.7 ± 9.7	62.9 ± 13.1	NS
FIGO				NS
II	1 (3 %)	1 (3.8 %)	0 (0 %)	
III	31 (94 %)	24 (92.4 %)	7 (100 %)	
IV	1 (3 %)	1 (3.8 %)	0 (0 %)	
Histopathology				NS
Serous	20 (60.7 %)	15 (57.7 %)	5 (71.4 %)	
Endometrioid	8 (24.2 %)	8 (30.8 %)	0 (0 %)	
Mucinous	1 (3 %)	1 (3.8 %)	0 (0 %)	
Clear cell	4 (12.1 %)	2 (7.7 %)	2 (29.6 %)	
Grading				NS
G1	1 (3 %)	1 (3.8 %)	0 (0 %)	
G2	15 (45.5 %)	13 (50 %)	2 (29.6 %)	
G3	17 (51.5 %)	12 (44.2 %)	5 (71.4 %)	

## Results

The optimal cytoreduction defined as less than 1.0 cm residual disease was achieved in all patients including the following: 26 patients (78.7 %) with no macroscopic residual disease, 4 patients (12.1 %) with the largest residual tumor less than 0.5, and 3 patients (9.1 %) with 0.5 cm to less than 1.0 cm residual disease.

The rectosigmoid resection was the most common surgical procedure (*n* = 27). The right hemicolectomy was performed in two patients, and the left hemicolectomy was also performed in three patients. In one case, the right hemicolectomy was accompanied by the rectosigmoid resection.

In 32 cases, the colon was reanastomosed using the end-to-end or side-to-side anastomosis stapler. One patient had two stapler anastomoses and required a protective colostomy. In one case, the presence of obstruction was the reason for a colostomy. The details of surgical procedures are shown in Table 2.

**Table 2:** Surgical procedures in patients with ovarian cancer

Surgical technique	Number
Sigmoid or rectosigmoid resection	28 (84.8 %)^a^
Right hemicolectomy	3 (9.1 %)^a^
Left hemicolectomy	3 (9.1 %)
Stapler anastomosis	32(97 %)^a^
Colostomy	2 (6.1 %)^a^
Maximum tumor diameter (cm)	11.3 (±5.4)^b^
Optimal debulking <1.0 cm (*n*)	34 (100 %)
No macroscopic residual tumor	26 (78.8 %)
Residual tumor <0.5 cm	4 (12.1 %)
Residual tumor 0.5–<1.0 cm	3 (9.1 %)
Time of operation (min)	209.4 (±35.0)^b^
Time of hospitalization (days)	9 (±4.5)^b^
Intraoperative blood loss (ml)	964.7 (±566.8)^b^

^a^One case subjected to a multiple procedure

^b^Values are mean ± *SD*

The postoperative period was uneventful in most patients. Neither fever nor deep vein thrombosis was observed in any case after the operation. One patient required reoperation due to gastric ulcer perforation. Abnormal wound healing was observed in three patients, but only one case required resuturing (Table 3).

**Table 3:** Perioperative complications

Complication	Number (%)
Heavy intraoperative bleeding (>1000 mL)	5 (15.5 %)
Fever	0 (0 %)
Abnormal wound healing	3 (9.1 %)
Anastomosis leak	0 (0 %)
Deep vein thrombosis	0 (0 %)
Gastric ulcer perforation	1 (3 %)
Prolonged hospitalization (>10 days)	5 (15.2%)

The median follow-up time was 656.9 ± 332.2 days (175–1312 days). The relapse was observed in seven patients (21.2 %). Five of them presented intraperitoneal spread. Local relapse in the pelvis was diagnosed in two cases. The mean progression-free survival (PFS) time was 411.0 ± 209.9 days. Four patients with the relapse died. The mean overall survival was 775.8 ± 521.4 days in this group. The detailed outcome is presented in Table 4. No deaths were observed in the group without the relapse during observation.

**Table 4:** Characteristics of patients with relapse

Patient number	Type of relapse	PFS (days)	Death	OS (days)	Follow-up (days)
1	Spread	334	Yes	523	523
2	Spread	252	Yes	675	675
3	Spread	236	Yes	375	375
4	Local	462	Yes	1538	1538
5	Local	854	No		1009
6	Spread	356	No		883
7	Spread	383	No		463
Mean		411.0		775.8	780.9
SD		209.9		521.4	404.0

In the whole group of optimal operated patients (residual disease <1.0 cm), the risk of relapse was significantly higher in subjects who had the macroscopic residual tumor left during the primary operation (57.1 vs. 11.5 %, *P* = 0.035). The size of primary ovarian tumor that infiltrated the bowel was another predictor of relapse. The maximum tumor diameter was significantly larger (14.9 ± 6.7 vs. 10.3 ± 4.7 cm, *P* = 0.047) in patients who developed the relapse. There was no association between the incidence of relapse and the initial CA 125 level or time from the operation to the onset of chemotherapy. Serum Ca125 level, patient’s age, the duration of operation, and perioperative blood loss did not differ in subjects with no and minimal (less than 1.0 cm) residual disease after the primary surgery (Table 5).

**Table 5:** Perioperative outcomes

	No relapse group (*N* = 26)	Relapse group (*N* = 7)	*P*
Maximum tumor diameter (cm)	14.9 ± 6.7	10.3 ± 4.7	0.047
Initial CA 125 (U/l)	642.7 ± 763.8	801.6 ± 1510.0	NS
Time from the operation to the onset of chemotherapy (days)	20.7 ± 5.2	19.7 ± 4.5	NS
No macroscopic residual tumor after operation	23 (88.5 %)	3 (42.9 %)	0.023
	No residual disease group (*N* = 27)	Minimal residual disease group (*N* = 7)	*P*
Initial CA 125 (U/l)	548.4 ± 713.0	1088.7 ± 1425.4	NS
Duration of operation (min)	206.9 ± 32.0	229.3 ± 35.2	NS
Perioperative blood loss (ml)	900.0 ± 494.1	1242.9 ± 793.4	NS

Values are mean ± SD

## Discussion

Cytoreductive surgeries performed in patients with advanced-stage ovarian cancer are among the most challenging in gynecologic oncology. Since the publication by Griffiths showing that survival is improved if the diameter of residual tumor implants after debulking does not exceed 15 mm, achieving the optimal cytoreduction became the main goal of surgery [10]. At present, the bulk of residual tumor is considered a main prognostic factor in patients treated with adjuvant chemotherapy with platinum (till the last decade of the twentieth century) and, more recently, with platinum and paclitaxel [11]. With time, the mass of residual tumor that still allows for calling surgery “radical” or “optimal” has been reduced, and at present, surgery is called “optimal” when no macroscopic tumor foci are left [4, 5, 12]. If possible, no tumor implants larger than 10 mm should be present at the end of surgery [13]. This strategy sometimes requires multiorgan resections. Most commonly, the parts of the digestive tract are resected, especially the large bowel. The reports on such procedures from the beginning of the twenty-first century showed promising results. In 2003, Bristow and coworkers published the results of surgery in 31 patients with stage IIIb and IV disease in whom rectosigmoid resection with stapler anastomosis was performed [14]. The radical cytoreduction defined as no tumor residues larger than 10 mm was achieved in 87.1 % of patients. There was no perioperative mortality, and an average blood loss was 700 ml. Anastomotic dehiscence occurred in one patient. The overall rate of large (dehiscence, bleeding) and small (fever, wound infection, pneumonia) complications was 12.9 and 35.5 %, respectively. Obermair and coworkers reported 65 cytoreductive surgeries with rectosigmoid resection and end-to-end anastomosis [15]. The optimal cytoreduction was achieved in 48 patients. The complications included the following: one bowel fistula (1.5 %), two cases of anastomotic dehiscence (3.1 %), two cases of ileus (3.1 %), 14 cases of wound infection (21.5 %), and five thromboembolic complications (7.7 %). Three patients were reoperated [15]. One patient died immediately after the surgery. Chia et al. reported on the results of surgery in 38 patients with advanced-stage ovarian cancer [16]. The most often performed operative procedure in this group of patients was resection of the sigmoid and rectum (76.3 %). Colostomy was performed in 61 % of patients. Optimal cytoreduction was achieved in 71 % of patients. Perioperative complications included one anastomotic dehiscence (2.6 %), one enteric fistula (2.6 %), and two interloop abscesses (5.3 %). Reoperation was required in three patients. During 30 days after the surgery, three patients died [16]. All aforementioned authors were unanimous that cytoreduction with bowel resection, most often of the sigmoid and rectum, gives good results and the rate of perioperative complications is acceptable. However, in the last mentioned paper, a high rate of colostomies brings attention. In 2007, we reported our initial experience in performing rectosigmoid resection or colectomy in patients with FIGO stage III and IV ovarian cancer [9]. Our series from 2007 consisted of 39 patients treated in a single center. We were able to achieve a radical cytoreduction (macroscopic implants <10 mm) in 32 patients (82 %). There were three (7.6 %) cases of anastomotic dehiscence, and one patient developed intestinal obstruction (2.5 %). The average blood loss was 1120 ml, and the average surgery time was 175 min. There were no perioperative deaths. Colostomy was performed in 36.4 % of 39 patients. This relatively high rate in early series of patients may be expected to be reduced with growing increased experience. In comparison with this paper, a significant reduction can be stated in the percentage of ostomy with increasing team experience.

In 2012, Peiretti and coworkers published the results of the multicenter study which was conducted between 1998 and 2008 and included 238 patients with carcinoma of the ovary, fallopian tube, and peritoneum in whom rectosigmoid resection was performed as a part of cytoreductive surgery [17]. Stapler anastomosis was performed in 98 % of patients. Ileostomy was performed in two (0.8 %) and colostomy in five (2 %) of the patients. Complete cytoreduction was achieved in 41 % of the patients. Anastomotic dehiscence occurred in nine patients (3 %) [17].

Anastomotic dehiscence (AD) is one of most serious complications related to bowel surgery, especially in rectosigmoid resection with low anastomosis. The incidence of this complication is between 2.8 and 23 % for resections of colonic cancer and between 0.8 and 6.8 % for gynecologic cancers [14, 15, 18, 19]. A lower rate of complications after gynecologic surgeries may be due to less common performance of the so-called low resections. The mortality of AD ranges from 7.3 to 16 %, and approximately one third of deaths related to colorectal surgery is due to AD [20, 21]. The factors contributing to a higher risk of AD include protracted surgery (surgery time longer than 2 h), blood transfusion, and short distal segment of anastomosis. It was suggested that relieving ileo- or colostomy may be used as a protective measure against this complication. On the other hand, overall rate of complications associated with en-block resection of the uterus and adnexa with tumor-infiltrated rectosigmoid without protective ileostomy is low, around 2 % [22]. Therefore, ileostomy should not be performed on a routine basis but only in specific situations such as a lack of bowel preparation or presence of factors worsening the prognosis, for instance, the necessity to perform more than one anastomosis [23]. Similarly, Hartmann’s procedure should be reserved only for patients with significant comorbidities or as “salvage procedure” [24].

Thorough preoperative work-out is a prerequisite of optimal cytoreduction. Well-selected imaging studies may help to avoid unnecessary surgery in patients with large unresectable foci of tumor. MDCT has established itself as an excellent tool for preoperative assessment of patients with ovarian cancer. The recent papers confirm high agreement of MDCT with both intraoperative and pathologic assessment of resectability and clinical grading of ovarian cancer [25]. Magnetic resonance imaging with diffusion-weighted imaging is very efficient in demonstrating the infiltration of adjacent organs and small peritoneal implants [26, 27]. High percentage of complete and optimal cytoreduction in our group confirmed the usefulness of MDCT or MRI with diffusion-weighted imaging during qualification for debulking surgery.

Several papers have addressed the issue of influence of extended surgery on survival. One of the first papers was published by Scarabelli and coworkers [28]. Two-year survival reached 100 % for patients without macroscopic tumor after surgery and 77 % for patients with foci of 1 cm in diameter. None of the patients with residual disease larger than 2 cm had survived 2 years [28]. According to the results of Takahashi and coworkers, the 5-year cumulative survival rate was 60.8 % in patients without residual disease and 0 % in patients with macroscopic disease <1 and >1 cm [29]. What is interesting about this report is also the observation that cumulative 5-year survival in patients undergoing primary radical cytoreductive surgery with bowel resection was 62.2 %, while that in patients operated after neoadiuvant chemotherapy was only 13.9 % [29]. Later retrospective studies also show promising results. Arora analyzed a group of 203 women with FIGO stage IIIc and IV ovarian cancer operated during a 10-year period [30]. In 51 patients from this group, optimal cytoreduction was achieved with bowel resection. The median follow-up time was 84 months (28–159). Two-year disease-free survival was reached by 63 % of patients. Considering that those results pertain to the patients with the most advanced-stage ovarian cancer, they must be perceived as satisfying. In an aforementioned paper by Priretti and coworkers, the recurrence occurred in 50 % of 238 patients during the study period [17]. However, only 5 % of them showed a relapse at the level of the pelvis whereas 8 % presented with abdominal recurrence associated with pelvic disease as well. The median overall survival time among patients with complete cytoreduction was 72 months compared with 42 months among the rest of the patients (*P* = 0.002) [17].

## Conclusions

As presented in the article, our outcomes and other authors’ observations indicate that debulking surgery with bowel resection in patients with advanced ovarian cancer brings good results. Complications connected with bowel surgery are to be accepted. The interesting thing is that a primary bowel tumor size was a predictor of relapse.

## Abbreviations

AD: anastomotic dehiscence
FIGO: The International Federation of Gynecology and Obstetrics
MDCT: multi-detector computed tomography
MRI: magnetic resonance imaging
PFS: progression-free survival
OS: overall survival

## References

[1] Didkowska J, Wojciechowska U, Zatonski W (2013). Cancer in Poland 2011.

[2] De Angelis R, Sant M, Coleman MP, Francisci S, Baili P, Pierannunzio D (2014). Cancer survival in Europe 1999-2007 by country and age: results of EUROCARE-5—a population-based study. Lancet Oncol.

[3] Verleye L, Ottevanger PB, Van der Graaf W, Reed NS, Vergote I, Gynaecological Cancer Group (GCG) of European Organisation for Research and Treatment of Cancer (EORTC) (2009). EORTC-GCG process quality indicators for ovarian cancer surgery. Eur J Cancer.

[4] Wimberger P, Lehmann N, Kimmig R, Burges A, Meier W, Du Bois A (2007). Prognostic factors for complete debulking in advanced ovarian cancer and its impact on survival. An exploratory analysis of a prospectively randomized phase III study of the Arbeitsgemeinschaft Gynaekologische Onkologie Ovarian Cancer Study Group (AGO-OVAR). Gynecol Oncol.

[5] Eisenhauer EL, Abu-Rustum NR, Sonoda Y, Aghajanian C, Barakat RR, Chi DS (2008). The effect of maximal surgical cytoreduction on sensitivity to platinum-taxane chemotherapy and subsequent survival in patients with advanced ovarian cancer. Gynecol Oncol.

[6] Bristow RE, Santillan A, Diaz-Montes TP, Gardner GJ, Giuntoli RL, Meisner BC (2007). Centralization of care for patients with advanced-stage ovarian cancer: a cost-effectiveness analysis. Cancer.

[7] Salani R, Zahurak ML, Santillan A, Giuntoli RL, Bristow RE (2007). Survival impact of multiple bowel resections in patients undergoing primary cytoreductive surgery for advanced ovarian cancer: a case-control study. Gynecol Oncol.

[8] Estes JM, Leath CA, Straughn JM, Rocconi RP, Kirby TO, Huh WK (2006). Bowel resection at the time of primary debulking for epithelial ovarian carcinoma: outcomes in patients treated with platinum and taxane-based chemotherapy. J Am Coll Surg.

[9] Bidzinski M, Derlatka P, Kubik P, Ziolkowska-Seta I, Dańska-Bidzinska A, Gmyrek L (2007). The evaluation of intra- and postoperative complications related to debulking surgery with bowel resection in patients with FIGO stage III-IV ovarian cancer. nt J Gynecol Cancer.

[10] Griffiths CT (1975). Surgical resection of tumor bulk in the primary treatment of ovarian carcinoma. Natl Cancer Inst Monogr.

[11] Bristow RE, Tomacruz RS, Armstrong DK, Trimble EL, Montz FJ (2002). Survival effect of maximal cytoreductive surgery for advanced ovarian carcinoma during the platinum era: a meta-analysis. J Clin Oncol.

[12] Eisenkop SM, Spirtos NM, Lin WC (2006). “Optimal” cytoreduction for advanced epithelial ovarian cancer: a commentary. Gynecol Oncol.

[13] Chi DS, Eisenhauer EL, Lang J, Huh J, Haddad L, Abu-Rustum NR (2006). What is the optimal goal of primary cytoreductive surgery for bulky stage IIIC epithelial ovarian carcinoma (EOC)?. Gynecol Oncol.

[14] Bristow RE, Del Carmen MG, Kaufman HS, Montz FJ (2003). Radical oophorectomy with primary stapled colorectal anastomosis for resection of locally advanced epithelial ovarian cancer. J Am Coll Surg.

[15] Obermair A, Hagenauer S, Tamandl D, Clayton RD, Nicklin JL, Perrin LC (2001). Safety and efficacy of low anterior en bloc resection as part of cytoreductive surgery for patients with ovarian cancer. Gynecol Oncol.

[16] Chia YN, Tay EH, Cheong DM, Eu KW, Low J, Ho TH (2003). Bowel surgery for epithelial ovarian cancer—an early case series. Ann Acad Med Singapore.

[17] Peiretti M, Bristow RE, Zapardiel I, Gerardi M, Zanagnolo V, Biffi R (2012). Rectosigmoid resection at the time of primary cytoreduction for advanced ovarian cancer. A multi-center analysis of surgical and oncological outcomes. Gynecol Oncol.

[18] Peeters KC, Tollenaar RA, Marijnen CA, Klein Kranenbarg E, Steup WH, Wiggers T (2005). Risk factors for anastomotic failure after total mesorectal excision of rectal cancer. Br J Surg.

[19] Yeh CY, Changchien CR, Wang JY, Chen JS, Chen HH, Chiang JM (2005). Pelvic drainage and other risk factors for leakage after elective anterior resection in rectal cancer patients: a prospective study of 978 patients. Ann Surg.

[20] McArdle CS, McMillan DC, Hole DJ (2005). Impact of anastomotic leakage on long-term survival of patients undergoing curative resection for colorectal cancer. Br J Surg.

[21] Wong NY, Eu KW (2005). A defunctioning ileostomy does not prevent clinical anastomotic leak after a low anterior resection: a prospective, comparative study. Dis Colon Rectum.

[22] Mourton SM, Temple LK, Abu-Rustum NR, Gemignani ML, Sonoda Y, Bochner BH (2005). Morbidity of rectosigmoid resection and primary anastomosis in patients undergoing primary cytoreductive surgery for advanced epithelial ovarian cancer. Gynecol Oncol.

[23] Kalogera E, Dowdy SC, Mariani A, Weaver AL, Aletti G, Bakkum-Gamez JN (2013). Multiple large bowel resections: potential risk factor for anastomotic leak. Gynecol Oncol.

[24] Seah DW, Ibrahim S, Tay KH (2005). Hartmann procedure: is it still relevant today?. ANZ J Surg.

[25] Kyriazi S, Kaye SB, DeSouza NM (2010). Imaging ovarian cancer and peritoneal metastases—current and emerging techniques. Nat Rev Clin Oncol.

[26] Michielsen K, Vergote I, Op de Beeck K, Amant F, Leunen K, Moerman P (2014). Whole-body MRI with diffusion-weighted sequence for staging of patients with suspected ovarian cancer: a clinical feasibility study in comparison to CT and FDG-PET/CT. Eur Radiol.

[27] Levy A, Medjhoul A, Caramella C, Zareski E, Berges O, Chargari C (2011). Interest of diffusion-weighted echo-planar MR imaging and apparent diffusion coefficient mapping in gynecological malignancies: a review. J Magn Reson Imaging.

[28] Scarabelli C, Gallo A, Franceschi S, Campagnutta E, De G, Giorda G (2000). Primary cytoreductive surgery with rectosigmoid colon resection for patients with advanced epithelial ovarian carcinoma. Cancer.

[29] Takahashi O, Sato N, Miura Y, Ogawa M, Fujimoto T, Tanaka H (2005). Surgical indications for combined partial rectosigmoidectomy in ovarian cancer. J Obstet Gynaecol Res.

[30] Arora M, Saha S, Puthillath A, Sehgal R, Dutt N, Metz J (2005). Impact of radical bowel resection on survival in advanced epithelial ovarian cancer. J Clin Oncol.

